# Supplementary material for: The origin and adaptive evolution of domesticated populations of yeast from Far East Asia
Source: Nat Commun. 2018 Jul 12;9:2690. doi: 10.1038/s41467-018-05106-7 (PMC6043522; doi:10.1038/s41467-018-05106-7)
Supplement: Supplementary file 1 — Supplementary Information [file 41467_2018_5106_MOESM1_ESM.pdf]

Supplementary Information

The Origin and Adaptive Evolution of Domesticated Populations of Yeast from Far East Asia

Shou-Fu Duan, Pei-Jie Han, Qi-Ming Wang, Wan-Qiu Liu, Jun-Yan Shi, Kuan Li, Xiao-Ling Zhang, Feng-Yan Bai*

State Key Laboratory of Mycology, Institute of Microbiology, Chinese Academy of Sciences, Beijing 100101, China

***Corresponding author, e-mail: baify@im.ac.cn.

The Supplementary Information contains

- Supplementary Figures 1 - 10
- Supplementary Notes 1 - 5
- Supplementary References

Supplementary Figure 1

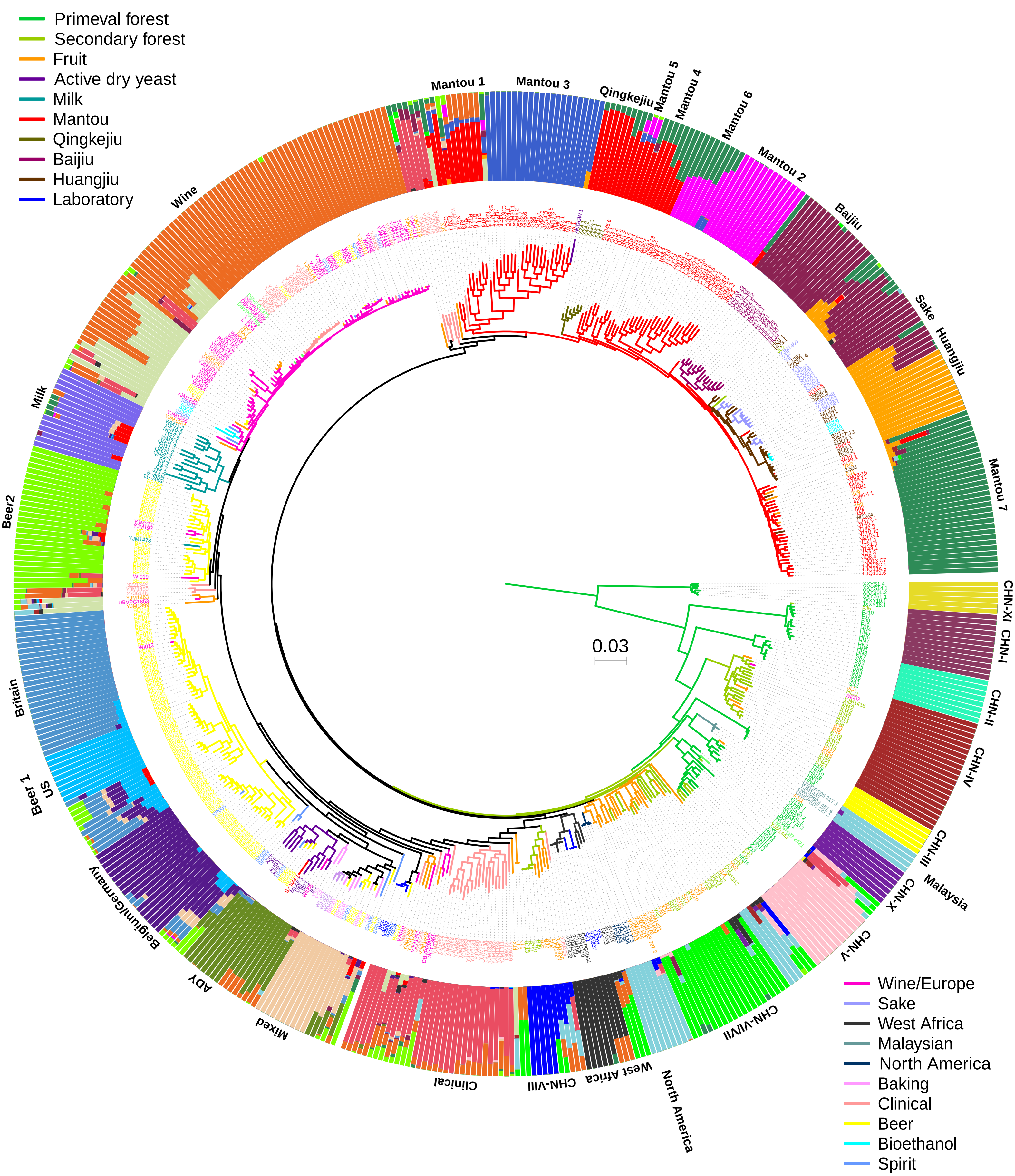

Supplementary Figure 2

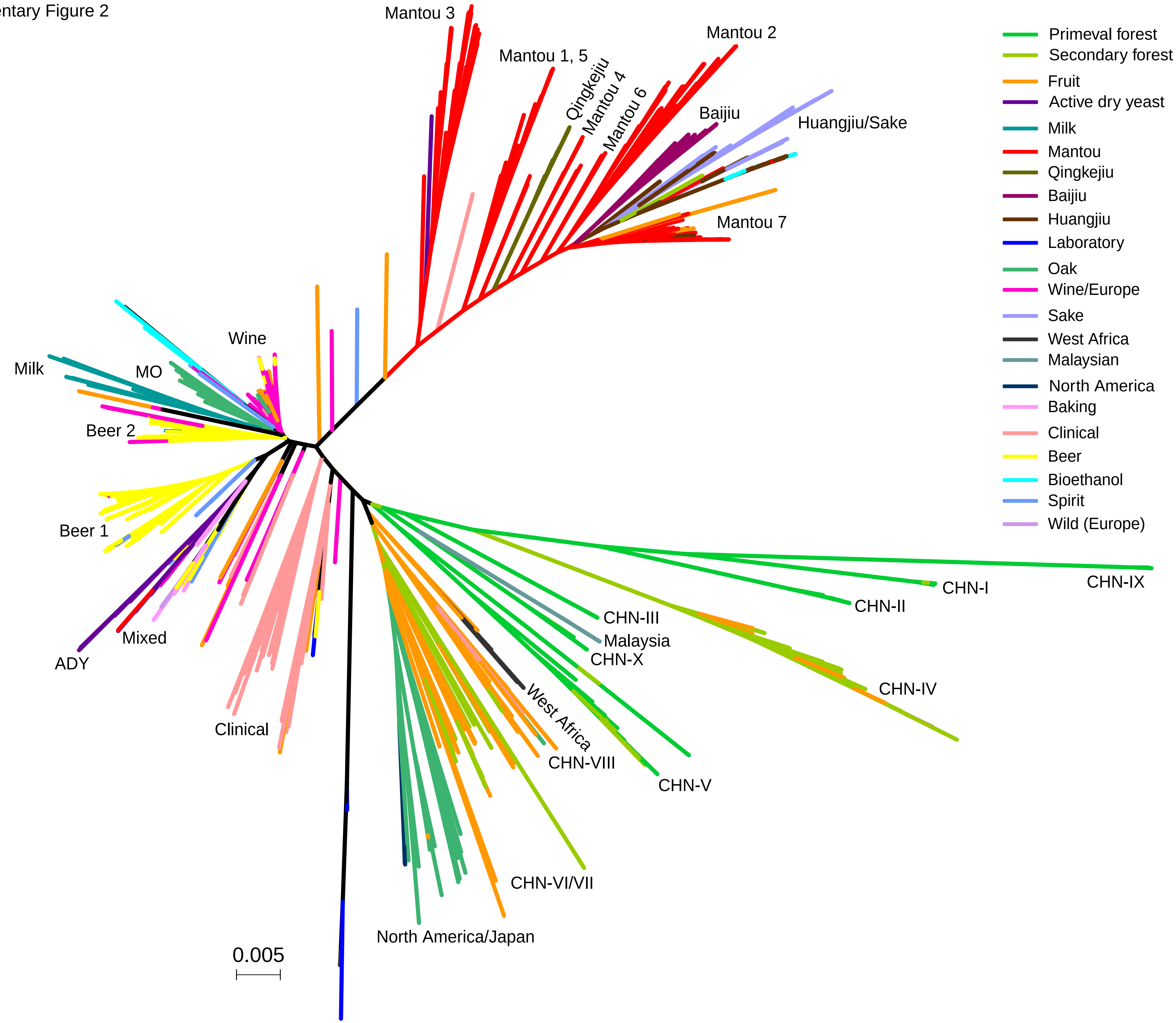

Supplementary Figure 3

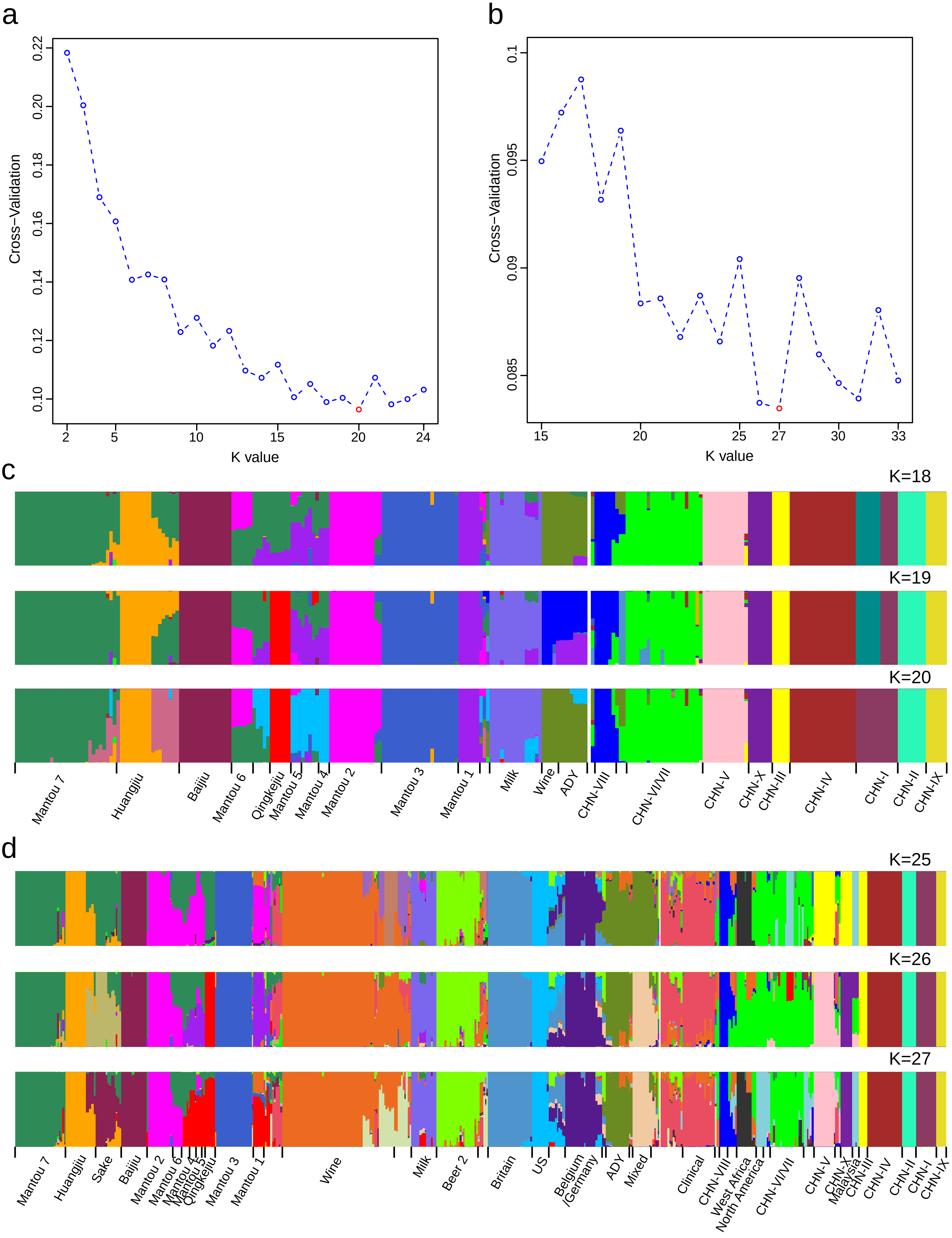

Supplementary Figure 4

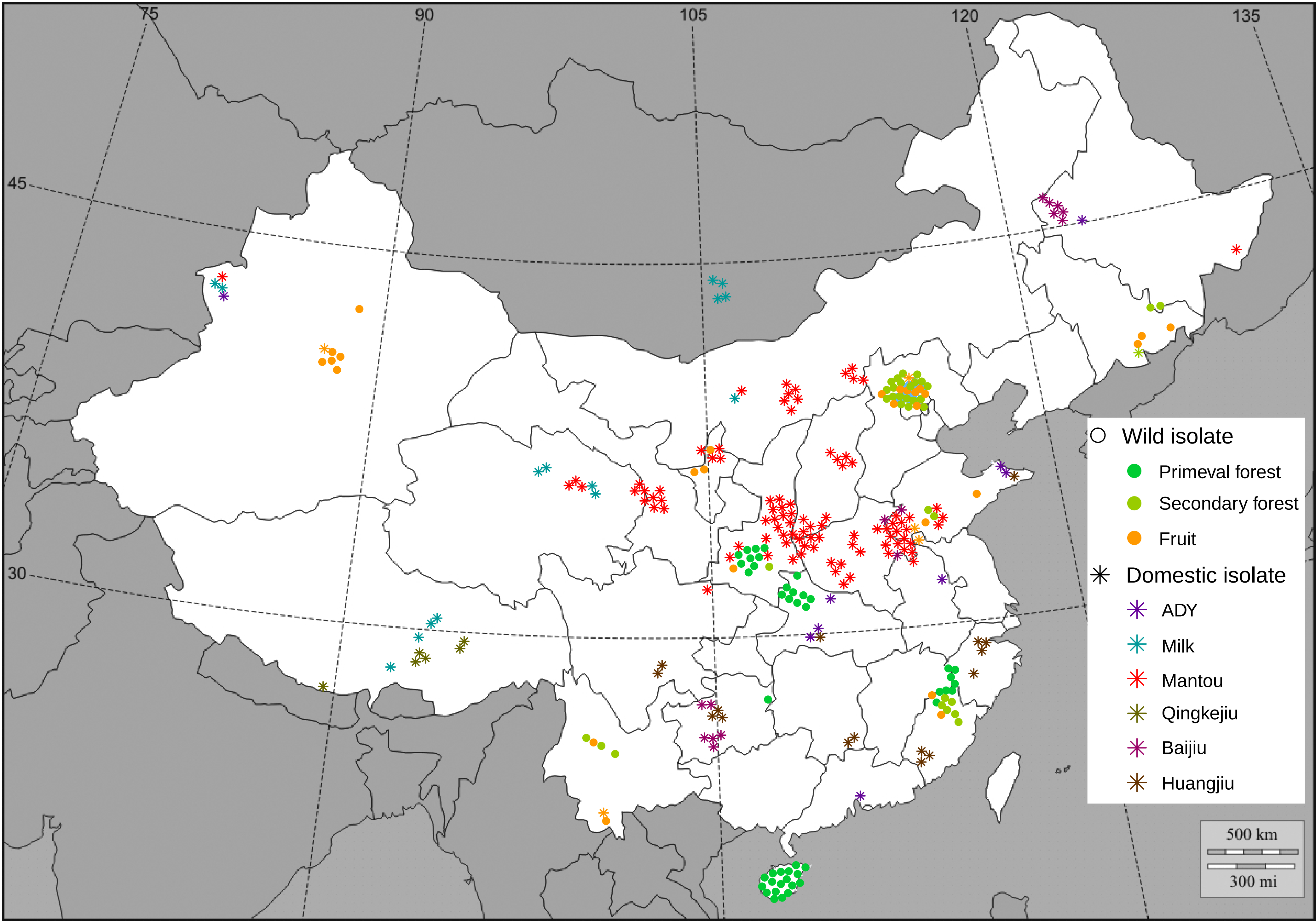

Supplementary Figure 5

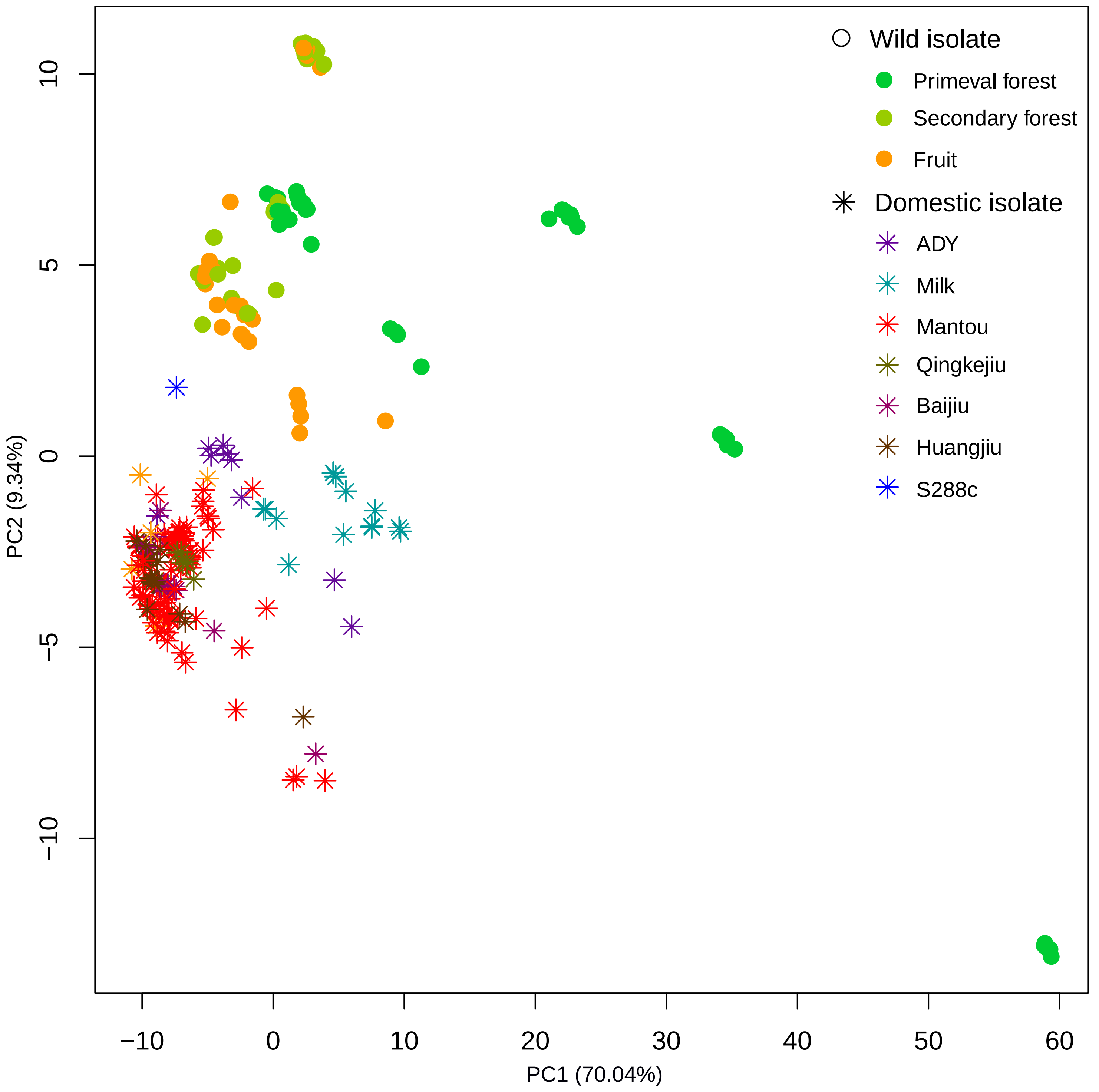

Supplementary Figure 6

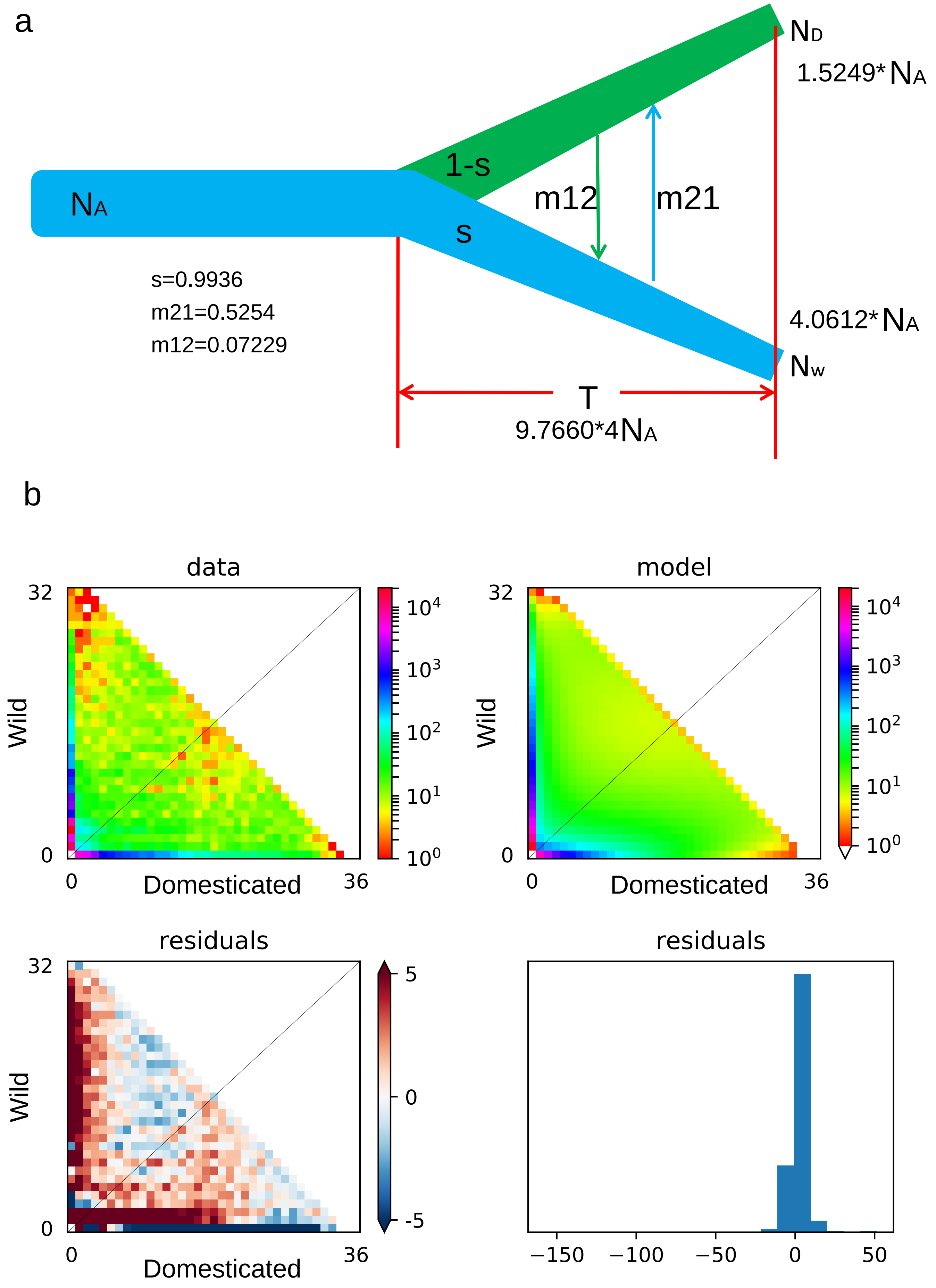

Supplementary Figure 7

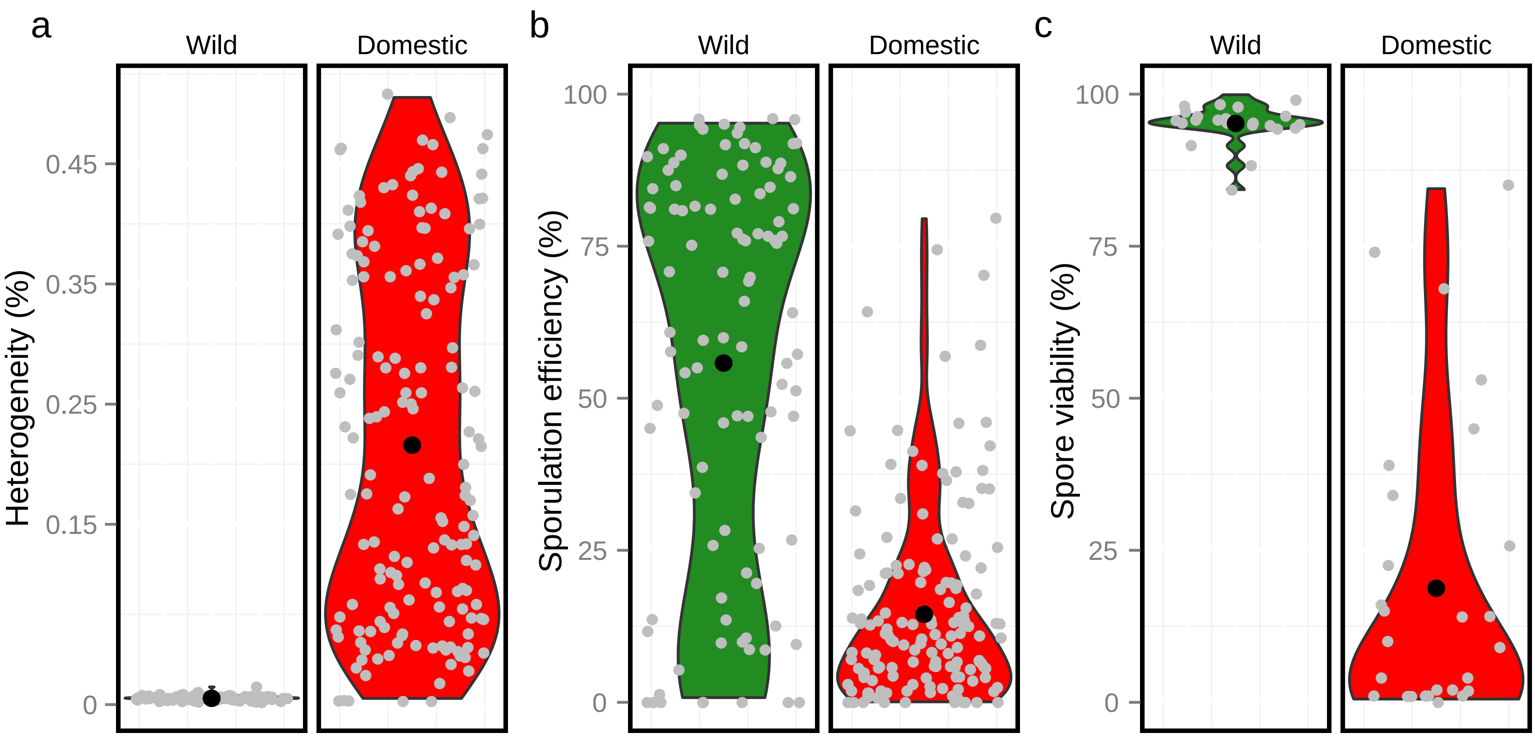

Supplementary Figure 8

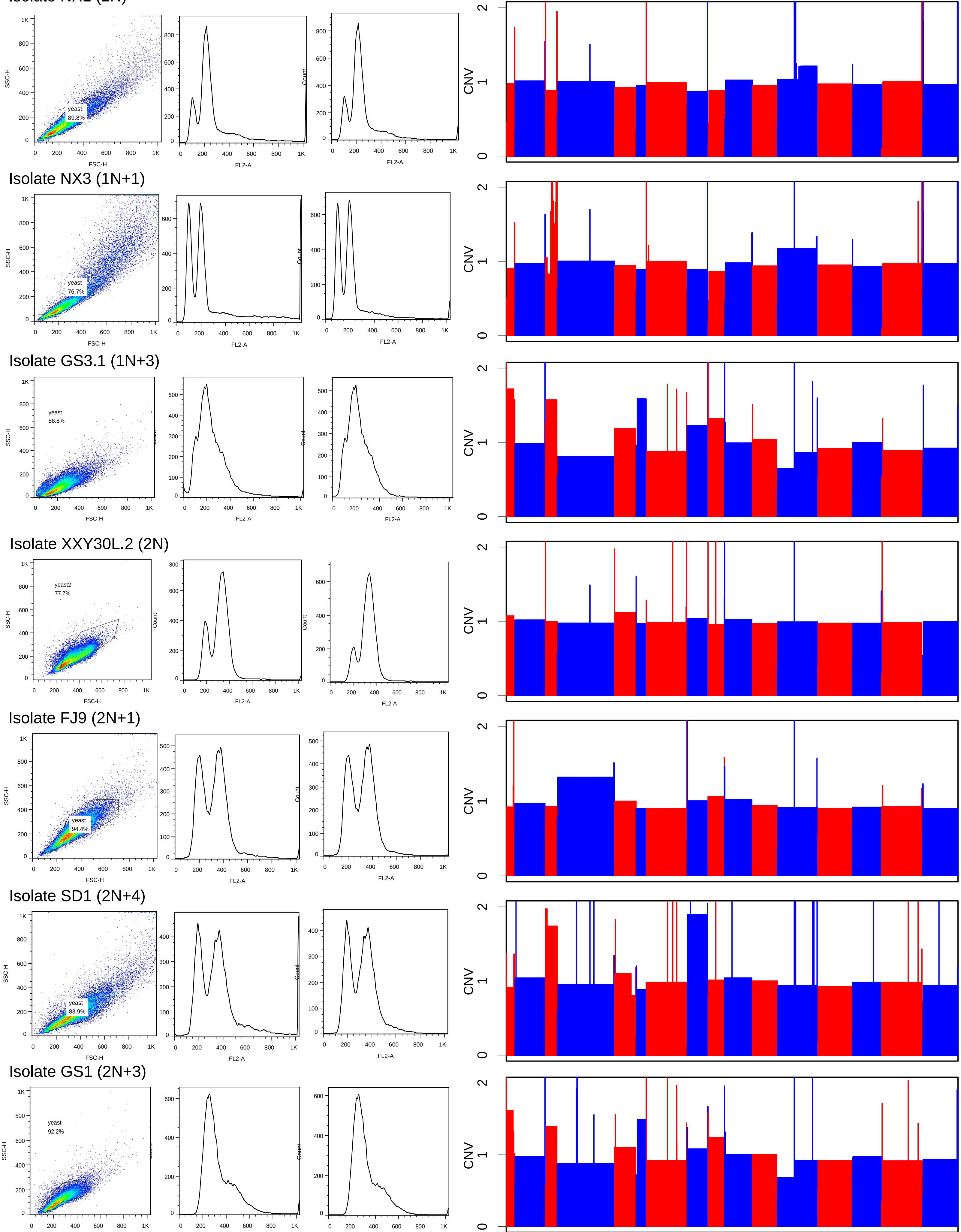

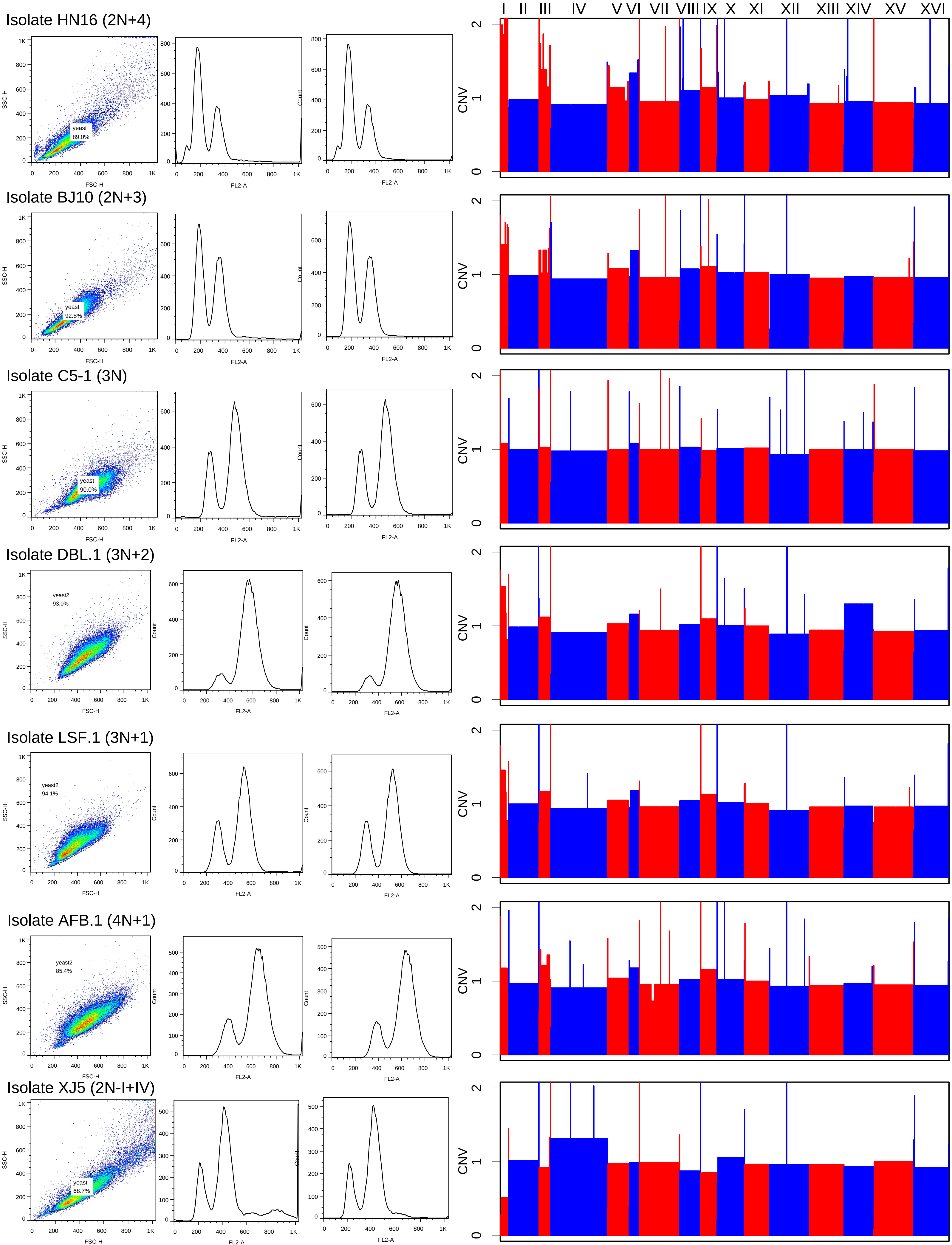
Isolate HN16 (2N+4)
Isolate BJ10 (2N+3)
Isolate C5-1 (3N)
Isolate DBL.1 (3N+2)
Isolate LSF.1 (3N+1)
Isolate AFB.1 (4N+1)
Isolate XJ5 (2N-I+IV)
I II III IV V VI VII VIII IX X XI XII XIII XIV XV XVI
CNV
SSC-H
FSC-H
FL2-A
Count
yeast
89.0%
92.8%
90.0%
yeast2
93.0%
94.1%
85.4%
68.7%

Supplementary Figure 9

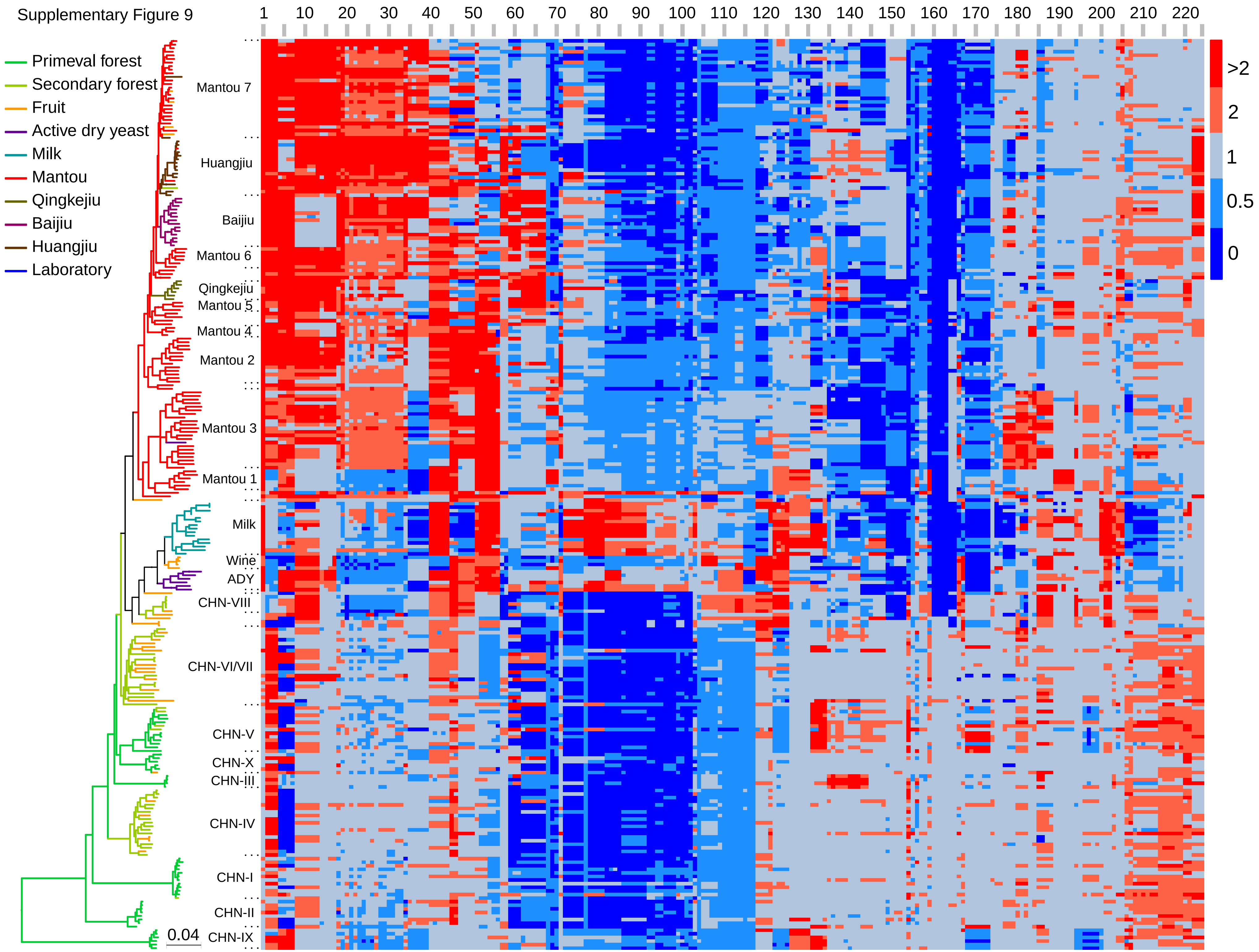

Supplementary Figure 10

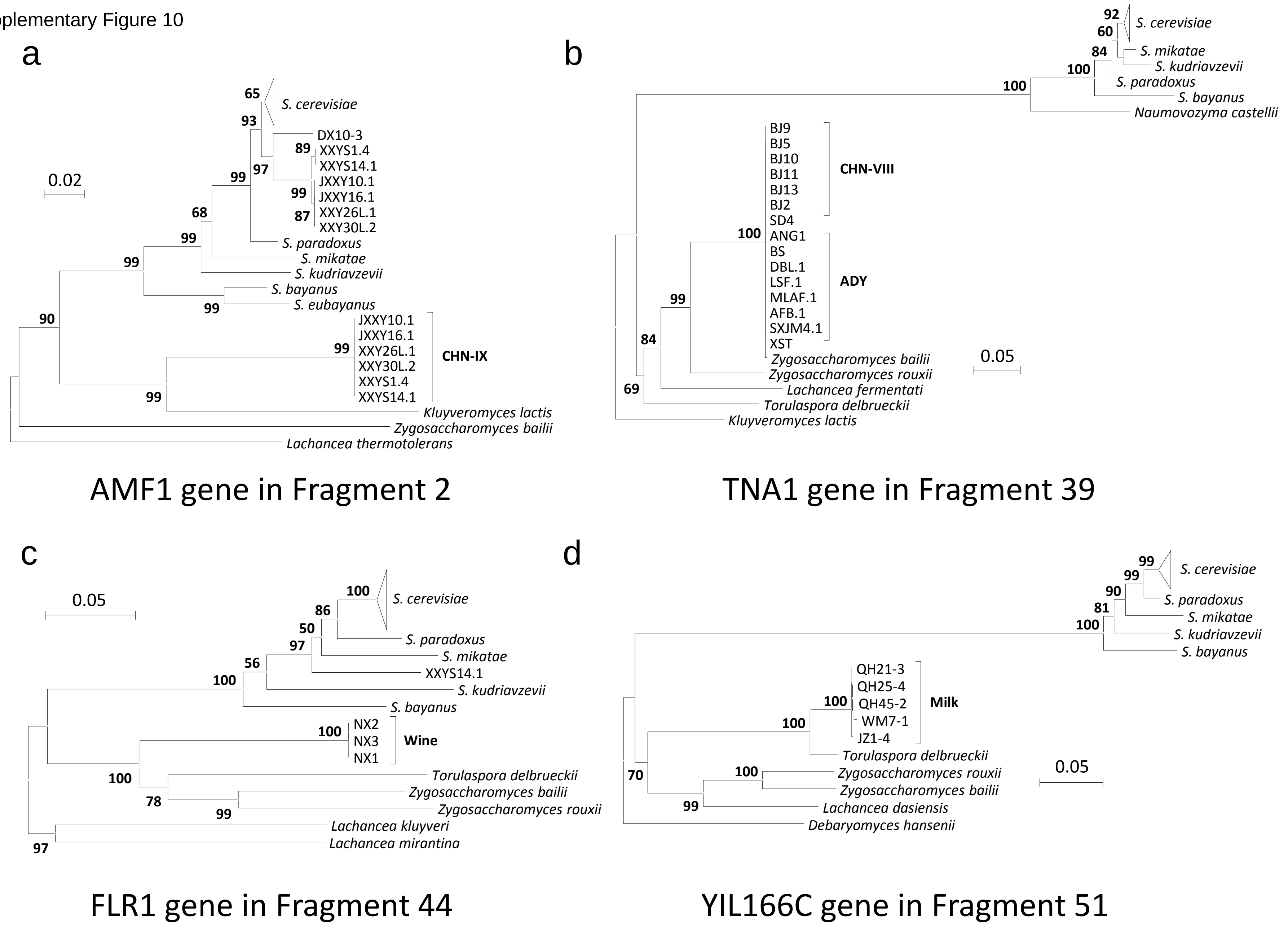

AMF1 gene in Fragment 2

TNA1 gene in Fragment 39

FLR1 gene in Fragment 44

YIL166C gene in Fragment 51

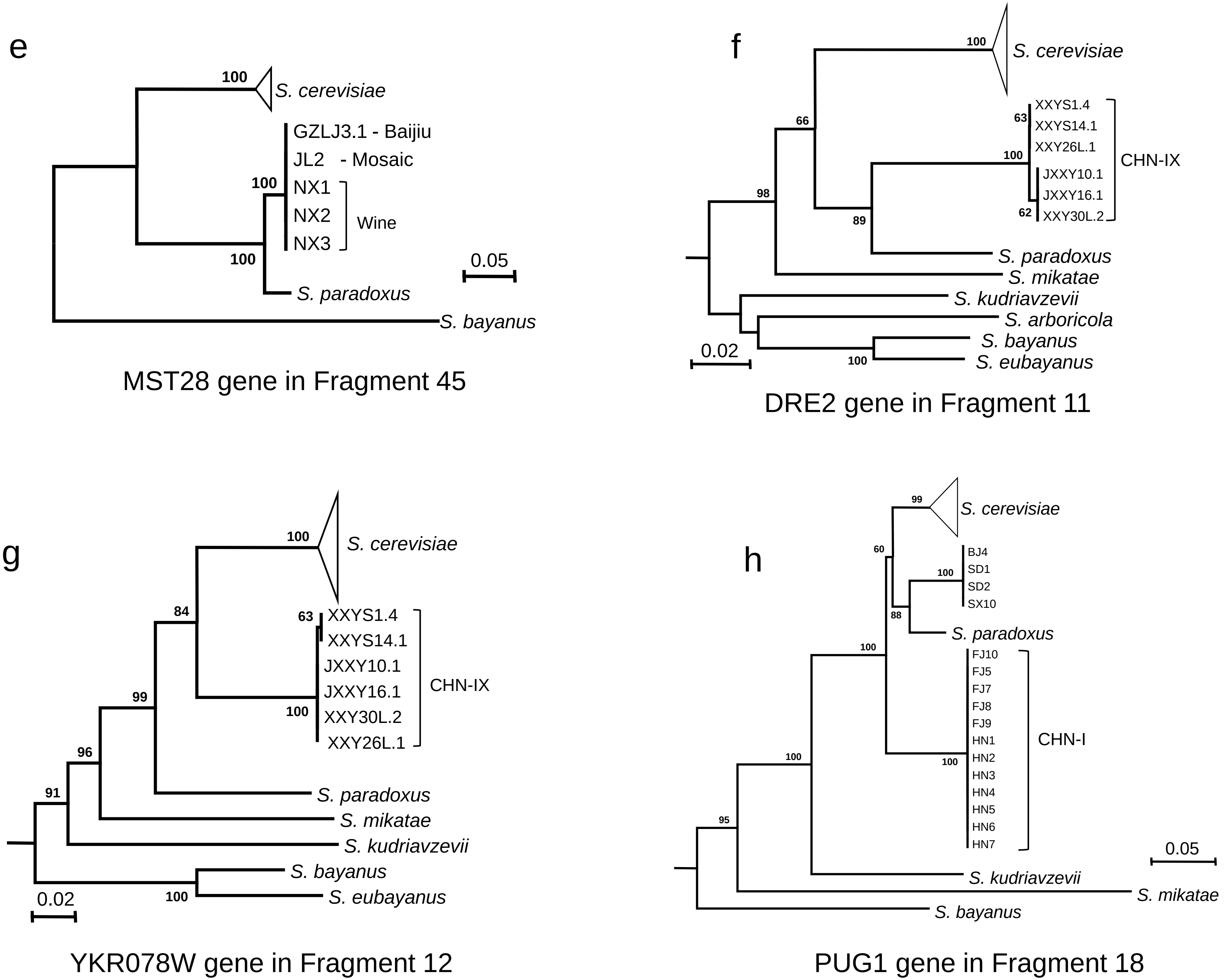
e
100
S. cerevisiae
GZLJ3.1 - Baijiu
JL2 - Mosaic
100
NX1
NX2
NX3
Wine
100
S. paradoxus
S. bayanus
0.05
MST28 gene in Fragment 45
f
100
S. cerevisiae
66
63
XXYS1.4
XXYS14.1
XXY26L.1
100
CHN-IX
JXXY10.1
JXXY16.1
62
XXY30L.2
98
89
S. paradoxus
S. mikatae
S. kudriavzevii
S. arboricola
S. bayanus
S. eubayanus
100
0.02
DRE2 gene in Fragment 11
g
100
S. cerevisiae
84
63
XXYS1.4
XXYS14.1
JXXY10.1
JXXY16.1
XXY30L.2
XXY26L.1
100
CHN-IX
99
96
91
S. paradoxus
S. mikatae
S. kudriavzevii
S. bayanus
S. eubayanus
100
0.02
YKR078W gene in Fragment 12
h
99
S. cerevisiae
60
BJ4
SD1
SD2
SX10
100
88
S. paradoxus
100
FJ10
FJ5
FJ7
FJ8
FJ9
HN1
HN2
HN3
HN4
HN5
HN6
HN7
CHN-I
100
100
95
S. kudriavzevii
S. mikatae
S. bayanus
0.05
PUG1 gene in Fragment 18

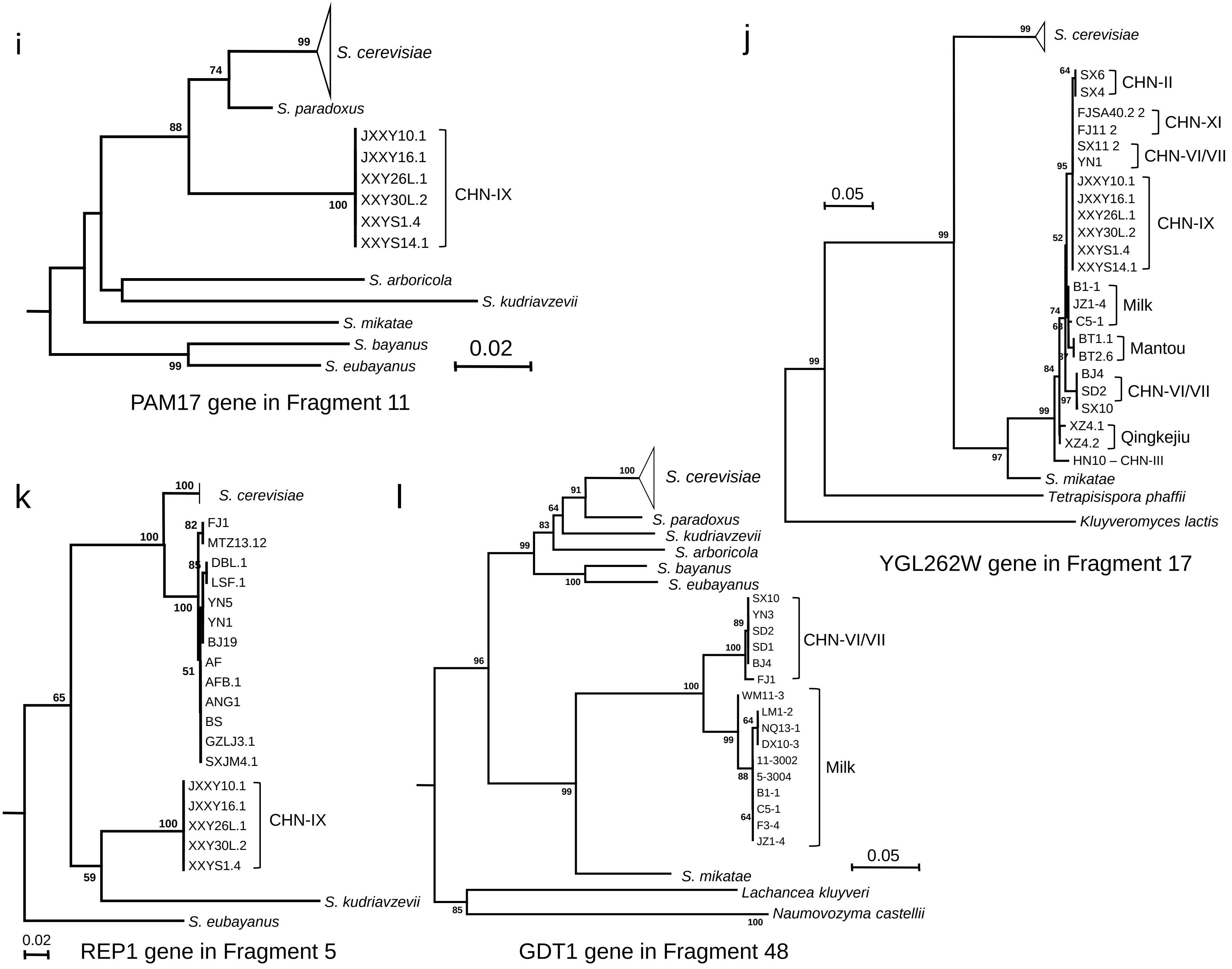
i
S. cerevisiae
S. paradoxus
JXXY10.1
JXXY16.1
XXY26L.1
XXY30L.2
XXYS1.4
XXYS14.1
CHN-IX
S. arboricola
S. kudriavzevii
S. mikatae
S. bayanus
S. eubayanus
0.02
PAM17 gene in Fragment 11
j
S. cerevisiae
SX6
SX4
CHN-II
FJSA40.2 2
FJ11 2
CHN-XI
SX11 2
YN1
CHN-VI/VII
0.05
JXXY10.1
JXXY16.1
XXY26L.1
XXY30L.2
XXYS1.4
XXYS14.1
CHN-IX
B1-1
JZ1-4
C5-1
Milk
BT1.1
BT2.6
Mantou
BJ4
SD2
SX10
CHN-VI/VII
XZ4.1
XZ4.2
Qingkejiu
HN10 – CHN-III
S. mikatae
Tetrapisispora phaffii
Kluyveromyces lactis
YGL262W gene in Fragment 17
k
S. cerevisiae
FJ1
MTZ13.12
DBL.1
LSF.1
YN5
YN1
BJ19
AF
AFB.1
ANG1
BS
GZLJ3.1
SXJM4.1
JXXY10.1
JXXY16.1
XXY26L.1
XXY30L.2
XXYS1.4
CHN-IX
S. kudriavzevii
S. eubayanus
0.02
REP1 gene in Fragment 5
l
S. cerevisiae
S. paradoxus
S. kudriavzevii
S. arboricola
S. bayanus
S. eubayanus
SX10
YN3
SD2
SD1
BJ4
FJ1
CHN-VI/VII
WM11-3
LM1-2
NQ13-1
DX10-3
11-3002
5-3004
B1-1
C5-1
F3-4
JZ1-4
Milk
0.05
S. mikatae
Lachancea kluyveri
Naumovozyma castellii
GDT1 gene in Fragment 48

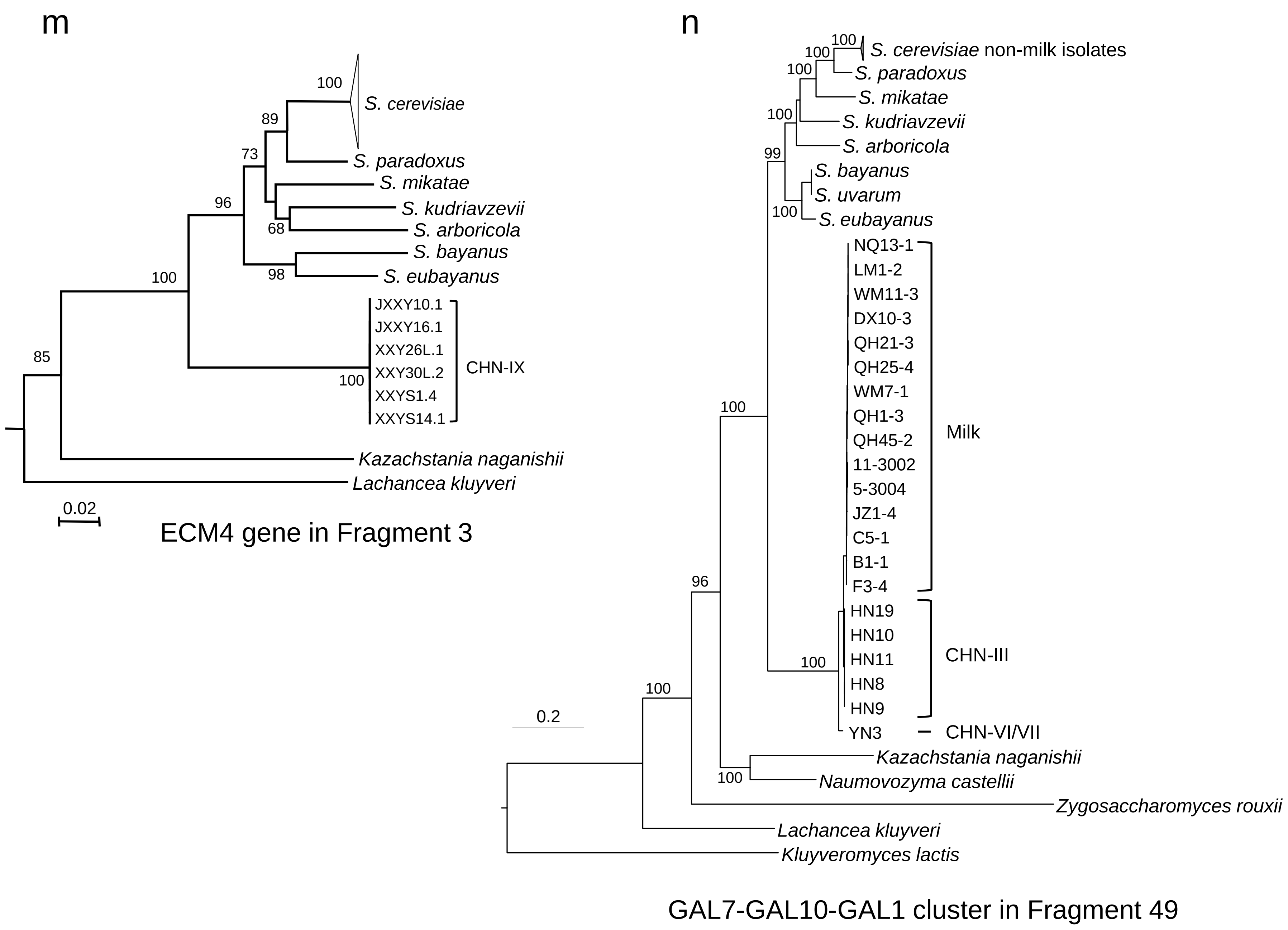
m
100
S. cerevisiae
89
73
S. paradoxus
S. mikatae
96
S. kudriavzevii
68
S. arboricola
S. bayanus
100
98
S. eubayanus
JXXY10.1
JXXY16.1
XXY26L.1
85
XXY30L.2
CHN-IX
100
XXYS1.4
XXYS14.1
Kazachstania naganishii
Lachancea kluyveri
0.02
ECM4 gene in Fragment 3
n
100
100
S. cerevisiae non-milk isolates
100
S. paradoxus
S. mikatae
100
S. kudriavzevii
S. arboricola
99
S. bayanus
S. uvarum
100
S. eubayanus
NQ13-1
LM1-2
WM11-3
DX10-3
QH21-3
QH25-4
WM7-1
100
QH1-3
QH45-2
Milk
11-3002
5-3004
JZ1-4
C5-1
B1-1
96
F3-4
HN19
HN10
CHN-III
100
HN11
HN8
100
HN9
0.2
YN3
CHN-VI/VII
Kazachstania naganishii
100
Naumovozyma castellii
Zygosaccharomyces rouxii
Lachancea kluyveri
Kluyveromyces lactis
GAL7-GAL10-GAL1 cluster in Fragment 49

Supplementary Figure legends

Supplementary Figure 1: Phylogeny and population structure inferred from a total of 554 *S. cerevisiae* isolates including 266, 38, 92 and 157 isolates sequenced in this study, Liti et al. (2009), Strope et al. (2015) and Gallone et al. (2016), respectively, and S288c. Phylogenetic trees were constructed by the maximum likelihood analysis based on 736,689 genome wide SNPs and rooted by lineage CHN-IX. Bootstrap support values to each lineage and major clade are 100% except for the Mangtou 7 lineage which is supported by 85% bootstrap resampling. Population structures were inferred using the ADMIXTURE program with K values being set to 27 determined by the minimum cross-validation error check. Isolates and terminal branches are colored according to ecological origins.

Supplementary Figure 2: Unrooted phylogenetic tree inferred from a total of 628 *S. cerevisiae* isolates including the isolates in Supplementary Fig. 1 and 51 oak isolates, 21 fermentation isolates (16 wine, two beer, and three sake isolates) and eight fruit isolates from the isolates sequenced in Almeida et al. (2015). Phylogenetic trees were constructed by the maximum likelihood analysis based on 206,810 SNPs. Terminal branches are colored according to ecological origins.

Supplementary Figure 3: Cross-validation (CV) tests for different K values based on (a) the dataset containing 266 Chinese *S. cerevisiae* isolates used in Figure 1 and (b) the dataset containing 554 worldwide isolates used in Supplementary Fig. 1; and comparisons of population structures inferred from the 266 isolate dataset (c) and the 554 isolate dataset (d) with K being set to different values as indicated. The program ADMIXTURE v1.23 was used for the CV tests and structure inference.

Supplementary Figure 4: Geographic distribution of the wild and domesticated *S. cerevisiae* isolates sampled in China and Mongolia.

Supplementary Figure 5: Principal component analysis (PCA) of genome wide SNPs from wild and domesticated isolates of *S. cerevisiae*.

Supplementary Figure 6: Sketch map of the best-fit Isolation-with-Migration model with exponential growth (IM) and corresponding joint allele frequency spectrum for representative wild and domesticated isolates of *S. cerevisiae*. (a) Sketch map depicting the evolution pattern of the IM model with population demographic parameters estimated based on this model. N_A, N_W and N_D represent the effective population sizes of the ancestral, wild and domesticated populations, respectively; s, the fraction of the ancestral population entered into the wild group; m12, migration rate from the domesticated to the wild group; m21, migration rate from the wild to the domesticated group; T, the split time. (b) Joint allele frequency spectrum for the wild and domesticated populations of *S. cerevisiae* and the optimal fitting of the data to the IM model. The upper panel shows the folded joint allele frequency spectrums of the actual data (left) and expected under the IM model (fight) using 97,895 non-coding SNPs. X and Y axes represent the number of strains in the wild and domesticated groups, respectively. Each entry is colored by the logarithm of the number of sites in it, according to the scale shown. The lower panel shows the plots (left) and histograms (right) of the residuals resulting from fitting the actual data to the IM model. The residuals represent the normalized difference between the IM model and actual data for each bin in the spectrum (red indicates that the model predicts too many SNPs in that bin and blue that the model predicts too few).

Supplementary Figure 7: Differences in heterozygosity expressed as the ratio of heterozygous SNPs to the consensus genome size of each isolate (a), sporulation efficiency (b) and spore viability (c) between the wild and domesticated populations of *S. cerevisiae*. The data shown in (a) and (b) were from 106 wild and 160 domesticated isolates and those in (c) were from 23 wild and 29 domesticated isolates with high sporulation efficiency.

Supplementary Figure 8: Flow cytometry and chromosome copy-number variation of representative isolates with different ploidies and chromosome amplification or deletion patterns.

Supplementary Figure 9: Copy-number variation (CNV) of genes in the wild and domesticated isolates of *S. cerevisiae*. Genes are numbered at the top of the heat map and their names are given in Supplementary Data 6 in the same order. Isolates are represented by terminal branches in the phylogenetic tree constructed from the maximum likelihood analysis on genome wide SNPs and are colored according to their ecological origins. Heat map colors reflect different degrees of gene duplication (red shades) or deletion (blue shades) from the basal level (grey) according to the scale on the right with strain S288c as the reference. The exact relative values of CNV are given in Supplementary Data 6.

Supplementary Figure 10: Phylogenetic trees constructed from neighbor-joining analyses based on amino acid sequences of proteins coded by genes harbored in representative introgression or horizontal gene transfer (HGT) fragments in S. cerevisiae isolates, showing sources of these alien fragments. Fragment numbers correspond to those in Supplementary Data 7.

Supplementary Notes

Supplementary Note 1: Detailed sources of fermentation-associated isolates

The sources of the fermentation-associated isolates include sourdough and fermenting dough for the production of Mantou (steamed bread) collected from families in countryside areas in different provinces of China; *Daqu* (fermentation starters) and fermenting grains for industrial production of various styles of Baijiu (Chinese distilled liquors made from sorghum), Huangjiu (rice wine) and Qingkejiu (highland barley wine); and fermented cow, yak, mare and goat milk and milk grains from local families in remote regions including Tibet, Xinjiang, Qinhai and Inner Mongolia in West and Northwest China. In addition, four isolates from fermented dairy products collected from Mongolia were included (Supplementary Data 1). *Daqu* (or *Koji* called in Japan) is mainly made from wheat and rice husk (with or without peas or barley) which are usually molded into bricks with 35-40% water and spontaneously fermented for about one month for the enrichment different microorganisms for saccharification and fermentation of sorghum, rice and highland barley for the production of Baijiu, Huangjiu and Qingkejiu, respectively. The temperatures within Daqu bricks change from ambient temperature to 40-50°C, 50-60°C or 60-70°C during the fermentation process, depending on different types[1]. For the production of Baijiu and Qingkejiu, steamed sorghum or highland barley grains are mixed with powdered Daqu (and with or without fermented grains from the last patch) and fermented in pits or jars in solid-state with about 55% water for about one month or more. The temperatures usually change from 10 to 30°C during the fermentation period and the alcohol content is usually 5-10% at the end of fermentation. For the production of Huangjiu, the raw material is steamed rice which is mixed with yeasts and *Daqu* and fermented in semi-liquid state in jars for about one month with the temperature changing from 10-33°C during the fermentation process. The alcohol content is usually 15-20% at the end of fermentation. The fermented dairy products sampled are all homemade and are various in raw materials as

mentioned above and fermentation conditions, but generally similar with Kefir[2] or Koumiss with low alcohol content (usually < 3%)[3]. The fermented food and beverages sampled are usually produced continuously in a year. Different batches of fermentation usually take place in the same pits or jars with residual fermented materials from the last batches.

Supplementary Note 2: Additional genes showing significant CNV

In addition to the genes discussed in the main text, a considerable number of other genes showing significant CNV are worth noting. Genes associated with stress response, including *AQY1* (encoding an aquaporin), *SGE1* (encoding a multiple drug-resistance protein acting as an extrusion permease), *SEO1* (associated with ethionine sulfoxide resistance), some *PAU* genes which probably help the yeast to cope with anaerobiosis[4,5], and the *ARR* gene cluster show a clear trend of expansion in the domesticated population. The *ARR* gene cluster containing three contiguous genes (*ARR1*, *ARR2* and *ARR3*) involves esistance to arsenic compounds[6,7] and is duplicated in the majority of domesticated isolates, except in the Baijiu and Mantou 1 lineages (Supplementary Data 6, Fig. 3). This cluster is also duplicated in the wild isolates from secondary forest and fruit in lineages CHN-VIII and Wine which are closely related the domesticated population.

Among the seven *AAD* genes which are putative aryl-alcohol dehydrogenase genes and probably involved in oxidative stress response[8], five (*AAD3*, *AAD6*, *AAD10*, *AAD15*, and *AAD16*) showed a clear trend of deletion in domesticated lineages (Supplementary Data 5), being in agreement with the prediction of their redundancy by Delneri et al., (1999)[8]. However, their maintenance in almost all wild isolates and expansion in wild isolates from secondary forests and fruit imply that they are functional genes for *S. cerevisiae* to live in the wild, especially in secondary forests and fruit. Similarly, *RDS1* which is located in chromosome III neighboring *AAD3* and encodes a putative zinc cluster transcription factor involved in conferring resistance to

cycloheximide[9] is deleted in most domesticated isolates but maintained in most wild isolates and even duplicated in wild isolates from secondary forests and fruit in lineages CHN-VIII and Wine. The duplication of the *AAD* genes and *RDS1* is probably due to the adaptation to environments with cycloheximide or similar antifungals or pesticides which are used for the control of plant pathogens. This trait is no longer required in fermentation environments and thus the genes related are deleted or contracted.

In addition to the *MAL* genes which are discussed in the main text, a considerable number of genes associated with sugar transportation and metabolisms are expanded in the majority of domesticated lineages. Among the genes in the hexose transporter (*HXT*) family, *HXT9* and *HXT12* showed a clear trend of expansion in domesticated isolates, especially in the solid-state fermentation lineages (Supplementary Data 6). *HXT12* was considered as a pseudogene in strain S288C because of its failure to transport hexoses when it was amplified from genomic DNA and overexpressed[10]. It is possibly a non-functional gene in the liquid state fermentation group and closely related fruit and laboratory isolates including S288c because it is lost or contracted in many isolates of this group. However, the remarkable expansion of this gene in the solid-state fermentation group suggests that it should be a functional gene in the populations of this group. Two genes *IMA3* and *IMA4* in the *IMA* isomaltase family encoding alpha-glucosidase[49], are simultaneously expanded in solid state fermentation isolates together with *HXT9* and *HXT12* (Supplementary Data 6).

A few genes associated with nitrogen source utilization, especially amino acid transportation, are deleted or contracted in domesticated lineages. *AGP3* (neighboring *AAD6* and *THI5* in chromosome VI) which encodes a low-affinity amino acid permease and may act to supply the cell with amino acids as nitrogen source in nitrogen-poor conditions, is deleted in most domesticated populations but retained in the Huangjiu and Baijiu lineages and all wild lineages. *VBA5* (neighboring *FLO10* and *NFT1*) encoding a vacuolar transporter for basic amino acids (*VBA*) involved in

amino acid uptake[11] is contracted in most domesticated lineages and in a wild lineage CHN-VIII from fruit and secondary forests but retained in domesticated lineages Baijiu and Mantou 7 and in the other wild lineages. *VBA3*, as a paralog of *VBA5*, is mainly deleted in the Milk population.

Some genes involved in maltose metabolism, including *MAL11* and *MAL1*3, are deleted mainly in the Milk lineage probably due to functional redundancy of these genes in the fermentation of milk. Two high-affinity glucose transporter genes *HXT6* and *HXT7* which locate adjacent to each other on Chromosome IV are also deleted in most isolates in the Milk lineage. The deletion of these two genes in the Milk lineage is unexpected because glucose is limited in fermenting milk and a previous study showed that *HXT6* and *HXT7* were duplicated in a laboratory population of *S. cerevisiae* due to selection in a glucose-limited environment[12].

The CNVs of the P-type ATPase Li^+/Na^+ pump-encoding *ENA* genes were observed in different *S. cerevisiae* isolates with different tolerant ability to Li^+ and Na^+ in previous studies[13,14]. We show here that *ENA6* is present in 236 (92.9%) of the 254 *S. cerevisiae* isolates analyzed (Supplementary Data 6). All the wild lineages from primeval forests and five domesticated lineages in the solid fermentation group contain only *ENA6*. *ENA1* began to rarely appear in lineage CHN-VI/VII from secondary forests and fruit and frequently occurred together with ENA2 and ENA5 in domesticated lineages ADY, Milk and Mantou 1 to Mantou 5 (Supplementary Data 6). This result clearly shows that *ENA6* is an ancestral gene in *S. cerevisiae* and *ENA1*, *ENA2*, *ENA5* were recently introgressed from *S. paradoxus*, validating the proposal of Strope et al. (2015)[14].

Supplementary Note 3: Detailed description of Introgression and HGT events

For the introgressed fragments from other species within the genus *Saccharomyces*, *S. paradoxus*, which is the closet relative of *S. cerevisiae*, is the dominant donor. The introgressed fragments are also usually lineage specific, distributing only in single or limited lineages. For examples, among the fragments with top matches from *S. paradoxus*, fragments 10-12 exist exclusively in lineage CH-IX; fragment 14 in CHN-I and CHN-IX; fragment 18 in CHN-I; and parts of fragments 27, 33 and 37 in CHN-II, CHN-III and CHN-V, respectively. A few fragments (no. 72, 73, 75, 76, 77, and 79) putatively from *S. paradoxus* are found widely in multiple lineages in the wild and domesticated populations. Phylogenetic analyses showed that fragments with more than 95% sequence identities with the homologs of *S. paradoxus* (e.g., nos. 10, 14, 45, 58 and 77) can be judged with confidence to have been introgressed from this species as shown in Supplementary Fig. 10e. However, the origins of the other fragments with top matches to *S. paradoxus* but with generally less than 95% sequence identities are uncertain. For examples, the *DRE2* gene in Fragment 11 (16,500 bp in length) detected in the CHN-IX lineage formed a branch closely related to but clearly separated from *S. paradoxus* (Supplementary Fig. 10f). The YKR078W gene harbored in Fragment 12 (3 kb in length) which also exclusively is found in the CHN-IX isolates is located on a branch between *S. cerevisiae* and *S. paradoxus* (Supplementary Fig. 10g), implying that this gene may represent an undescribed close relative species to the two species. The top matches of the 1-kb windows of fragment 18 which occurs exclusively in CHN-I, were mainly from *S. paradoxus* with less than 90% identities. Phylogenetic analyses based on the amino acid sequences of the gene *PUG1* contained in this fragment showed that the CHN-I isolates formed a branch basal to *S. cerevisiae* and *S. paradoxus* (Supplementary Fig. 10h). Similarly, the phylogeny of gene *PRM17* harbored in fragment 11 showed that this gene from the CHN-IX isolates also formed a branch basal to *S. cerevisiae* and *S. paradoxus* (Supplementary Fig. 10i). These

fragments differed from *S. paradoxus* and *S. cerevisiae* by 12.0%- 6.3% and 13.9%-11.5% nucleotide sequence divergence, respectively, beyond the maximum genome sequence divergence (4.5%) within *S. paradoxus*, suggesting the possible existence of an unknown or missing species or lineage basal to *S. cerevisiae* and *S. paradoxus* within the genus *Saccharomyces*.

Fragment 17 (2.5 kb in length) distributing in a few wild and domesticated lineages was probably introgressed from *S. mikatae*. The top matches of this fragment were mainly from *S. mikatae* with 98.1% to 99.0% sequence identities in the 1-kb mapping windows (Supplementary Data 7, Supplementary Fig. 10j). In addition to *S. paradoxus* and *S. mikatae*, *S. bayanus*, *S. kudriavzevii*, and *S. uvarum* were included in the top matches of some of the possibly introgressed fragments (Supplementary Data 7), but the sequence identities were quite low (usually less than 90%). The sources of these fragments are therefore uncertain. Phylogenetic analyses showed that some of them might be introgressed from unknown species or lineages within *Saccharomyces*. For examples, the *REP1* gene contained in fragment 5 distributing specifically in lineage CHN-IX and the *GDT1* gene harbored in fragment 48 distributing specifically in lineage Milk might from unknown species or lineages closely related with *S. kudriavzevii* and *S. mikatae*, respectively, as shown in the phylogenetic trees (Supplementary Fig. 10k-l).

It is worth noting that the top matches of a considerable number of the putative introgressed fragments were from various *S. cerevisiae* strains (Supplementary Data 7). They are regarded as representing possible introgression events based on the following considerations: 1) they usually occur in limited lineages or isolates; 2) they differ from the matched fragments in the reference *S. cerevisiae* strains by over 10% nucleotides; and 3) they differ from the matched fragments in the other majority of *S. cerevisiae* isolates sequenced in this study by over 7% nucleotides, significantly beyond the maximum inter-lineage genome sequence divergence of 1.6% as shown in Supplementary Data 3; and 4) phylogenetic analyses showed that some of them may

be introgressed from unknown sources or present transitional states. We also found that some *S. cerevisiae* strains sequenced in other studies share HGT or introgression fragments with our isolates sequenced in this study. For example, BLAST search through GenBank showed that Fragment 1 also exist in three *S. cerevisiae* isolates YJM1388, YJM1389 and YJM1592 sequenced in Strope et al. (2015)[14] with 100% identity. YJM1388 was from fermented tapioca in Malaya and YJM1389 and YJM1592 were from sewage in Thailand. They were all clustered in the Sake lineage. It will be interesting to perform a further systematic survey of HGT or introgression events in all the sequenced *S. cerevisiae* strains with different ecological and geographic origins in the future.

Supplementary Note 4: Additional phenotypes associated with ecology and genomic variations

A total of 54 (20.3%) of the isolates tested could utilize melibiose and these isolates mainly concentrated in wild lineages CHN-III (5/5, 100%), CHN-V (10/13, 76.9%) and CHN-X (3/7, 42.9%) and domesticated lineages Mantou 1 (6/6, 100%), Mantou 7 (7/29, 24.1%), Baijiu (6/15, 40%) and Huangjiu (11/16, 73.3%). With the exception of six isolates in the Milk lineage, all the wild and domesticated isolates tested grew well in raffinose and the melibiose positive isolates showed approximately doubled raffinose utilization efficiency as compared with the other raffinose positive isolates (Fig. 5, Supplementary Data 8). This can be explained because raffinose is firstly hydrolyzed into fructose and melibiose by ß-fructosidase and the isolates that could not utilize melibiose stop growing when the released fructose is used up. As expected, all the isolates unable to grow in melibiose have lost the *MEL* gene which is responsible for melibiose utilization, except for ten isolates (FJ6, GT39.1, GT99.1, HLJ4, HN18, HQ3.1, JQ9.3, SX9, SXJM6.6 and XZ4.1), which contain an intact *MEL* gene each but are unable to utilize melibiose. The cause of their failure to utilize melibiose remains to be revealed.

All the isolates tested grew well in sucrose, except six isolates in the Milk lineage. These isolates formed a subclade in the Milk lineage and contained only *SUC2* of the *SUC* gene family responsible for sucrose consumption[15]. We found that a deletion of a base ‘T’ at site 36 of the gene resulted in codon shift and disfunction of *SUC2* in these isolates. The mutation is probably due to relaxed selection pressure because of the absence of sucrose in the growth environment of these isolates. These six isolates are unable to utilize raffinose either (Supplementary Data 8).

For the tolerance to high temperatures (40°C and 41°C), lineage specific variations were observed. Among the wild lineages, the isolates in lineages CHN-I, CHN-II, CHN-III and CHN-IX from primeval forests were unable to grow at 40°C; while the majority of the isolates in lineages CHN-IV, CHN-V, CHN-VI/VII and CHN-VIII from fruit and secondary forests grew well at 40°C (Supplementary Data 8, Fig. 5). Among the domesticated lineages, the isolates in the Milk, ADY and Mantou 3 showed negative to weak growth at 40°C while the other domesticated lineages in the solid-state fermentation group usually grow well at this temperature. The isolates that grew relatively well at 41°C were concentrated in wild lineage CHN-VI/VII from secondary forests and fruit and in the domesticated solid-state fermentation group. The ethanol tolerance test showed that, in general, the domesticated isolates, especially those in the solid fermentation group, showed a trend of increased tolerance to 9% ethanol (Fig. 5, Supplementary Data 8).

Supplementary Note 5: Neutrality and selection tests

The McDonald-Kreitman (MK) test[16,17] was performed to detect genes subjected to different pressures of selection in different populations of *S. cerevisiae* recognized in this study. A gene dataset for the MK test was constructed following the requirements below: i) each gene was identified exactly as a single copy gene from assembled genomes; ii) only a gene with ≥ 90% amino acid identity and 80% amino acid coverage thresholds among different lineages was included, the genes showing exceptionally diverged sequences among different lineages or populations were

excluded; iii) only strains with genes that meet the requirements above were included, however, each gene set should cover at least 80% of the strains in a given group identified from the phylogenetic analysis to ensure the sampling sizes of each group. By setting six isolates (Y17217, UWOPS919171, UFRJ50816, YPS138, N44 and CBS432) from four lineages of *S. paradoxus* as the outgroup, we obtained a total of 4210 genes from the 266 isolates sequenced in this study which were eligible for calculating the numbers of synonymous (Ds) and non-synonymous (Dn) substitutions and the numbers of synonymous (Ps) and non-synonymous (Pn) polymorphisms using the EggLib tools[18]. Then we corrected for multiple hypothesis testing using the Benjamini-Hochberg method[19]. The genes subjected to positive and purifying selection and being neutral to selection detected and related parameters including the proportion of base substitutions fixed by natural selection (Alpha)[20], neutrality index (NI), and fixation index (F_{ST})[21] calculated using EggLib are listed in Supplementary Data 9a. The genes with Alpha values greater or smaller than zero significantly ($P <$ 0.05) were regarded as under positive or purifying selection. Otherwise they were regarded as being selectively neutral.

We found that a much higher fraction of genes were subjected to purifying selection in the domesticated population than in the wild population of *S. cerevisiae* by the MK test[17] (Supplementary Data 9). We detected a very limited number of genes evolving under positive selection in *S. cerevisiae* (Supplementary Data 9). However, we found that more than half (51.5%) of the genes subjected to the MK test demonstrated purifying selection in the domesticated population. In contrast, only 8.9% of the genes tested demonstrated purifying selection in the wild population and the remaining (91.0%) were consistent with a neutral model (Supplementary Data 9). Interestingly, Gene Ontology (GO) enrichment analysis detected significant enrichment of the class meiotic chromosome separation (GO:0051307) in the genes under purifying selection in the wild but not in the domesticated isolates

(Supplementary Data 9b), probably due to the reduced sexuality in the domesticated isolates.

Supplementary References

1 Jin, G., Zhu, Y. & Xu, Y. Mystery behind Chinese liquor fermentation. *Trends Food Sci. Tech.* **63**, 18-28, (2017).

2 Prado, M. R. *et al.* Milk kefir: composition, microbial cultures, biological activities, and related products. *Front. Microbiol.* **6**, 1177, (2015).

3 Mu, Z., Yang, X. & Yuan, H. Detection and identification of wild yeast in Koumiss. *Food Microbiol.* **31**, 301-308, (2012).

4 Luo, Z. & van Vuuren, H. J. Functional analyses of *PAU* genes in *Saccharomyces cerevisiae*. *Microbiology* **155**, 4036-4049 (2009).

5 Rachidi, N., Martinez, M. J., Barre, P. & Blondin, B. *Saccharomyces cerevisiae PAU* genes are induced by anaerobiosis. *Mol. Microbiol.* **35**, 1421-1430 (2000).

6 Maciaszczyk, E., Wysocki, R., Golik, P., Lazowska, J. & Ulaszewski, S. Arsenical resistance genes in *Saccharomyces douglasii* and other yeast species undergo rapid evolution involving genomic rearrangements and duplications. *FEMS Yeast Res.* **4**, 821-832 (2004).

7 Bergstrom, A. *et al.* A high-definition view of functional genetic variation from natural yeast genomes. *Mol. Biol. Evol.* **31**, 872-888 (2014).

8 Delneri, D., Gardner, D. C. & Oliver, S. G. Analysis of the seven-member *AAD* gene set demonstrates that genetic redundancy in yeast may be more apparent than real. *Genetics* **153**, 1591-1600 (1999).

9 Akache, B. & Turcotte, B. New regulators of drug sensitivity in the family of yeast zinc cluster proteins. *J. Biol. Chem.* **277**, 21254-21260 (2002).

10 Wieczorke, R. *et al.* Concurrent knock-out of at least 20 transporter genes is required to block uptake of hexoses in *Saccharomyces cerevisiae*. *FEBS Lett.* **464**, 123-128 (1999).

11 Shimazu, M. *et al.* Vba5p, a novel plasma membrane protein involved in amino acid uptake and drug sensitivity in *Saccharomyces cerevisiae*. *Biosci. Biotechnol. Biochem.* **76**, 1993-1995 (2012).

12 Brown, C. J., Todd, K. M. & Rosenzweig, R. F. Multiple duplications of yeast hexose transport genes in response to selection in a glucose-limited environment. *Mol. Biol. Evol.* **15**, 931-942 (1998).

13 Warringer, J. *et al.* Trait variation in yeast is defined by population history. *PLoS Genet.* **7**, e1002111 (2011).

14 Strope, P. K. *et al.* The 100-genomes strains, an *S. cerevisiae* resource that illuminates its natural phenotypic and genotypic variation and emergence as an opportunistic pathogen. *Genome Res.* **25**, 762-774 (2015).

15 Marques, W. L., Raghavendran, V., Stambuk, B. U. & Gombert, A. K. Sucrose and *Saccharomyces cerevisiae*: a relationship most sweet. *FEMS Yeast Res.* **16**, fov107 (2016).

16 Welch, J. J. Estimating the genomewide rate of adaptive protein evolution in *Drosophila*. *Genetics* **173**, 821-837 (2006).

17 McDonald & Kreitman. Adaptive protein evolution at the Adh locus in *Drosophila*. *Nature* **351**, 652-654 (1991).

18 De Mita, S. & Siol, M. EggLib: processing, analysis and simulation tools for population genetics and genomics. *BMC Genet.* **13**, 27 (2012).

19 Benjamini, Y. & Hochberg, Y. Controlling the false discovery rate: a practical and powerful approach to multiple testing. *J. R. Statist. Soc. B* **57**, 289-300 (1995).

20 Smith, N. G. & Eyre-Walker, A. Adaptive protein evolution in *Drosophila*. *Nature* **415**, 1022-1024 (2002).

21 Holsinger, K. E. & Weir, B. S. Genetics in geographically structured populations: defining, estimating and interpreting F(ST). *Nat. Rev. Genet.* **10**, 639-650 (2009).
