# Supplementary material for: The origin and adaptive evolution of domesticated populations of yeast from Far East Asia
Source: Nat Commun. 2018 Jul 12;9:2690. doi: 10.1038/s41467-018-05106-7 (PMC6043522; doi:10.1038/s41467-018-05106-7)
Supplement: Supplementary file 3 — Description of Additional Supplementary Files [file 41467_2018_5106_MOESM3_ESM.pdf]

Description of Additional Supplementary Files

File Name: Supplementary Data 1

Description: *Saccharomyces cerevisiae* isolates sequenced and their ecological and geographic origins.

File Name: Supplementary Data 2

Description: Genome sequence data and sporulation rates of *S. cerevisiae* isolates employed.

File Name: Supplementary Data 3

Description: Genetic diversity within and divergence between different groups, populations or lineages of *S. cerevisiae*.

File Name: Supplementary Data 4

Description: Proportions of shared polymorphisms among different populations and lineages of *S. cerevisiae* in China

File Name: Supplementary Data 5

Description: Ploidy and chromosome copy-number variations of *S. cerevisiae* isolates.

File Name: Supplementary Data 6

Description: Copy number variation (CNV) of genes between different populations or lineages of *S. cerevisiae* (a) and functional descriptions of the genes related grouped by GO categories (b).

File Name: Supplementary Data 7

Description: Introgression and horizontal gene transfer (HGT) events in wild and domesticated isolates of *S. cerevisiae* (a) and genes harbored in the HGT and introgression fragments (b).

File Name: Supplementary Data 8

Description: Phenotypic characterization of 266 *S. cerevisiae* isolates employed.

File Name: Supplementary Data 9

Description: Genes subject to positive and purifying selection detected by the MK test from wild and domesticated *S. cerevisiae* isolates (a) and GO enrichment analysis of genes subject to purifying selection (b).
